# Revisiting the Boundaries of GDMT Optimization: Beyond Adverse Effects and Into the Future of Personalized Heart Failure Care

**DOI:** 10.1002/clc.70187

**Published:** 2025-07-26

**Authors:** Ateeq Ur Rehman, Syed Muhammad Rayyan, Areeba Khan

**Affiliations:** ^1^ Ayub Medical College Abbottabad Pakistan

## Conflicts of Interest

The authors declare no conflicts of interest.

To the Editor,

We read with interest the study by Velasco et al. on tolerability and adverse effects (AEs) encountered during heart failure (HF) guideline‐directed medical therapy (GDMT) optimization [1]. The authors highlight an increasingly relevant challenge in HF management: real‐world implementation of GDMT often falls short of guideline recommendations. While the study sheds light on important aspects of AEs and patient characteristics influencing dose titration, several key limitations and oversights merit critical discussion.

First, the inclusion of only those patients who completed the optimization program introduces selection bias, possibly underestimating the true burden of titration‐limiting AEs. Patients unable to complete the program potentially due to more severe AEs or clinical decompensation were excluded from analysis. This risks portraying a more favorable safety and tolerability profile than might exist in broader clinical practice [1]. Additionally, the lack of a comparator group limits the ability to assess whether the program truly improved outcomes beyond natural progression or standard care.

Second, while older age and atrial fibrillation were identified as predictors of suboptimal beta‐blocker and RAAS inhibitor dosing, the effect sizes were modest (OR 1.04 for age) and potentially confounded by unmeasured variables such as frailty or social support [1]. Surprisingly, hypertension appeared protective, contradicting conventional expectations. This counterintuitive finding may reflect physiological buffering in hypertensive patients or clustering of other unmeasured favorable traits. We believe it suggests a need to explore vascular‐autonomic phenotyping in HF patients, an underexplored frontier.

Moreover, the focus on four common AEs, bradycardia, hypotension, hyperkalemia, and renal dysfunction while pragmatic, overlooks other frequent clinical barriers such as fatigue, dizziness, ACEi‐induced cough, polypharmacy, and most crucially, therapeutic inertia [2, 3]. Clinical inertia, the reluctance to intensify treatment despite suboptimal control, has been shown to significantly impede GDMT application, with over 50% of physicians reducing therapy based on anticipated rather than actual AEs [4].

The study's population was relatively young, with fewer comorbidities, and a notable “obesity paradox”: obese patients were more likely to reach target doses [1]. This reflects findings from the Swedish HF Registry, where obese patients had greater GDMT adherence, possibly due to physiological reserve or clinician bias [5]. This observation raises the question: should GDMT targets be individualized rather than uniform?

Importantly, the authors report no titration‐limiting AEs in patients on SGLT2 inhibitors, aligning with emerging evidence of their favorable safety profile and supporting their expanded use as foundational therapy in HFrEF [1, 6].

Future research should move beyond rigid dose targets to personalized, phenotype‐driven strategies incorporating frailty indices, biomarker‐guided titration, and technology‐enhanced monitoring. The STRONG‐HF trial, for example, demonstrated that rapid post‐discharge up‐titration with close follow‐up led to a 34% relative risk reduction in death or HF hospitalization despite an increase in minor AEs [7].

Velasco et al. rightly call for standardized AE definitions in GDMT research. We emphasize the need for standardized management algorithms as well as detailing when to pause, rechallenge, or continue therapy. Such frameworks can mitigate clinician hesitation and bridge the gap between guideline ideals and bedside realities.

In conclusion, while the authors have advanced the discourse on GDMT optimization, the path forward demands integration of clinical nuance, system‐level solutions, and individualized care. We commend their efforts and advocate for further investigation that dares to question assumptions and elevate patient‐centered precision in HF therapy.

## Author Contributions

All authors contributed equally to the preparation of the manuscript.

## Ethics Statement

The authors have nothing to report.

## Consent

The authors have nothing to report.

## Data Availability

The authors have nothing to report.

## References

[1] C. M. Velasco , G. Baksh , M. Haydo , H. Reesor , J. Boehmer , and O. Ali , “Tolerability and Adverse Effects in a Specialized Heart Failure Guideline‐Directed Medical Therapy Optimization Program,” Clinical Cardiology 48, no. 7 (2025): e70179, 10.1002/clc.70179. 40662449 PMC12261027

[2] S. J. Greene and G. C. Fonarow , “Clinical Inertia and Medical Therapy for Heart Failure: The Unintended Harms of ‘First, Do No Harm’,” European Journal of Heart Failure 23, no. 8 (2021): 1343–1345, 10.1002/ejhf.2283. 34184376

[3] P. M. Seferović , M. Polovina , C. Adlbrecht , et al., “Navigating Between Scylla and Charybdis: Challenges and Strategies for Implementing Guideline‐Directed Medical Therapy in Heart Failure With Reduced Ejection Fraction,” European Journal of Heart Failure 23, no. 12 (2021): 1999–2007, 10.1002/ejhf.2378. 34755422

[4] G. Savarese , F. Lindberg , R. M. Christodorescu , et al., “Physician Perceptions, Attitudes, and Strategies Towards Implementing Guideline‐Directed Medical Therapy in Heart Failure With Reduced Ejection Fraction. A Survey of the Heart Failure Association of the ESC and the ESC Council for Cardiology Practice,” European Journal of Heart Failure 26, no. 6 (2024): 1408–1418, 10.1002/ejhf.3214. 38515385

[5] C. Cappelletto , D. Stolfo , N. Orsini , et al., “Use of and Association Between Heart Failure Pharmacological Treatments and Outcomes in Obese Versus Non‐Obese Patients With Heart Failure With Reduced Ejection Fraction: Data From the Swedish Heart Failure Registry,” European Journal of Heart Failure 25, no. 5 (2023): 698–710, 10.1002/ejhf.2795. 36781199

[6] J. Harrington , G. C. Fonarow , M. S. Khan , et al., “Medication‐Attributable Adverse Events in Heart Failure Trials,” JACC: Heart Failure 11, no. 4 (2023): 425–436, 10.1016/j.jchf.2022.11.026. 36881395 PMC10084875

[7] A. Mebazaa , B. Davison , O. Chioncel , et al., “Safety, Tolerability and Efficacy of Up‐Titration of Guideline‐Directed Medical Therapies for Acute Heart Failure (STRONG‐HF): A Multinational, Open‐Label, Randomised, Trial,” Lancet 400, no. 10367 (2022): 1938–1952, 10.1016/S0140-6736(22)02076-1. 36356631

